# Characterization of Cyclodextrin/Volatile Inclusion Complexes: A Review

**DOI:** 10.3390/molecules23051204

**Published:** 2018-05-17

**Authors:** Miriana Kfoury, David Landy, Sophie Fourmentin

**Affiliations:** 1 Bioactive Molecules Research Laboratory, Doctoral School of Sciences and Technologies, Faculty of Sciences II, Lebanese University, Fanar, Lebanon; mirianakfoury@hotmail.com; 2 Unité de Chimie Environnementale et Interactions sur le Vivant (UCEIV), EA 4492, SFR Condorcet FR CNRS 3417, Université du Littoral-Côte d’Opale (ULCO), 59140 Dunkerque, France; landy@univ-littoral.fr

**Keywords:** cyclodextrin, fluorescence, formation constant, HPLC, ITC, NMR, phase solubility, SH-GC, TOC, UV-Visible, volatiles

## Abstract

Cyclodextrins (CDs) are a family of cyclic oligosaccharides that constitute one of the most widely used molecular hosts in supramolecular chemistry. Encapsulation in the hydrophobic cavity of CDs positively affects the physical and chemical characteristics of the guests upon the formation of inclusion complexes. Such a property is interestingly employed to retain volatile guests and reduce their volatility. Within this scope, the starting crucial point for a suitable and careful characterization of an inclusion complex is to assess the value of the formation constant (K_f_), also called stability or binding constant. This task requires the application of the appropriate analytical method and technique. Thus, the aim of the present paper is to give a general overview of the main analytical tools used for the determination of K_f_ values for CD/volatile inclusion complexes. This review emphasizes on the advantages, inconvenients and limits of each applied method. A special attention is also dedicated to the improvement of the current methods and to the development of new techniques. Further, the applicability of each technique is illustrated by a summary of data obtained from the literature.

## 1. Introduction

The field of supramolecular encapsulation using cyclodextrins (CDs) continues to grow [1]. The use of CD is particularly interesting with volatile compounds [2,3,4,5,6]. Indeed, encapsulation in CDs represents a feasible and efficient tool to retain and modulate the release of the encapsulated volatiles [7,8,9,10]. The volatile guests include mainly volatile organic compounds (VOCs), aroma and flavors, anesthetics, etc.

**Figure 1:**
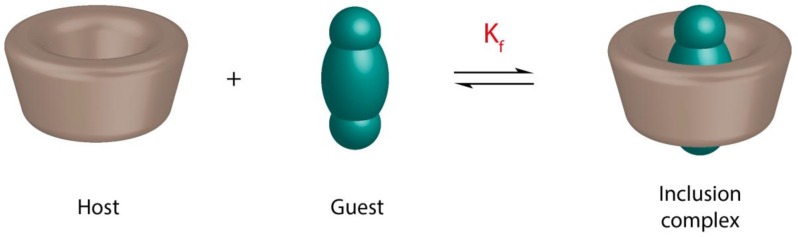
Schematic illustration of the formation of an inclusion complex between a cyclodextrin (host) and a guest.

The main feature of CDs is the formation of inclusion complexes with the guests (Figure 1). The most fundamental parameter in the quantitative analysis of the binding strength between the CD and guest is the determination of the formation constant (K_f_) of each inclusion complex. K_f_ values are determined mainly to answer two different types of questions. The first one, which deals with the encapsulation in an absolute mean, is, can a CD encapsulate the guest? The second question is a comparative one, that is, what is the binding strength? Which inclusion complex is more stable?

Although a wide number of analytical methods are available for the characterization of inclusion complexes in solution and a large progress in analytical methodologies has been accomplished [3,11], few could be applied to CD/volatile inclusion complexes. This is mainly due to the low aqueous solubility of most of the volatile compounds.

In general, the applied methods can be divided into four main groups: spectroscopic methods: UV-Visible (UV-Vis) spectroscopy [12,13,14], fluorescence spectroscopy [15,16] and nuclear magnetic resonance spectroscopy (NMR) [2]; chromatographic methods: static headspace coupled to the gas chromatography (SH-GC) [7,8,9,17,18,19,20,21] and high-performance liquid chromatography (HPLC) [22], calorimetric methods: isothermal titration calorimetry (ITC) [23,24,25] and solubility studies [10,26,27,28,29,30,31,32,33,34,35]. Lately, a new Total Organic Carbon (TOC) method was also developed [36].

The aim of this paper is to provide an overview of the research that has explored the assessment of K_f_ values for CD/volatile guest inclusion complexes. The experimental procedures and the obtained data are outlined in more detail. Finally, the advantages and limitations of the applied methods are critically discussed.

## 2. Characterization of Cyclodextrin Inclusion Complexes

**Figure 2:**
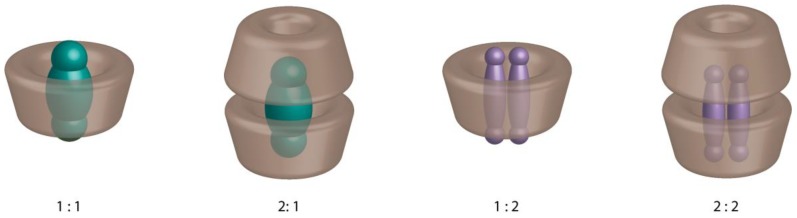
Schematic representation of the main stoichiometries of the inclusion complexes.

The initial step in the characterization of an inclusion complex is the determination of the stoichiometry and formation constant (K_f_) values. If various stoichiometries could be observed in the literature (Figure 2), most of the inclusion complexes present a 1:1 (CD:guest) stoichiometry [2].

All the inclusion complexes described in this review possess a 1:1 stoichiometry. In this case, the K_f_ could be expressed as:

(1) $$K_{f}=\frac{\left[\mathrm{Inclusion}\;\mathrm{complex}\right]}{\left[\mathrm{Host}\right]\left[\mathrm{Guest}\right]}=\frac{\left[\mathrm{CD}/G\right]}{\left[\mathrm{CD}\right]\left[G\right]}=\frac{\left[\mathrm{CD}/G\right]}{\left({\left[\mathrm{CD}\right]}_{T}-\left[\mathrm{CD}/G\right]\right)\left({\left[G\right]}_{T}-\left[\mathrm{CD}/G\right]\right)}=\frac{\left[\mathrm{CD}/G\right]}{{\left[\mathrm{CD}\right]}_{T}{\left[G\right]}_{T}-{\left[\mathrm{CD}\right]}_{T}\left[\mathrm{CD}/G\right]-\left[\mathrm{CD}/G\right]{\left[G\right]}_{T}+{\left[\mathrm{CD}/G\right]}^{2}}$$

with [G]_T_ the initial guest concentration and [G] the free guest concentration. [CD]_T_ the initial CD concentration and [CD] the free CD concentration.

Various treatments could be used for the resolution of this equation. These treatments will be described in the corresponding sections of the analytical techniques.

Titration experiments using a constant concentration of a species (titrate) and increasing amounts of the other species (titrant) are generally employed. Nevertheless, some alternative approaches suitable for low soluble compounds, such as volatiles, have been also developed, in particular competitive methods and phase solubility studies.

### 2.1. Volatilization Method

The first method used for the determination of K_f_ between a CD and a volatile guest was based on the fact that volatile compounds could be driven out from an aqueous solution to gaseous phase by an inert gas bubbling at a constant flow rate in the aqueous solution [37,38,39]. The volatilization rate of the guest is supposed to be decreased in the presence of the CD. This decrease will depend on the strength of the association with the CD. This method was applied firstly to determine the K_f_ for iodine and then of aliphatic, cyclic or aromatic hydrocarbons [38,39]. The obtained results are listed in Table 1.

This technique, developed by Sanemasa’s group, has used home-made device and was no longer used after the 1990s. From this time, Sanemasa’s group has worked on the developement of static headspace-gas chromatography (SH-GC) methods.

### 2.2. Chromatographic Methods

#### 2.2.1. Static Headspace-Gas Chromatography

Static headspace coupled to gas chromatography (SH-GC) is widely employed to analyze volatile compounds in various fields [40,41]. This technique allows the quantification of a volatile present in a gaseous phase in contact and equilibrium with a condensed phase (liquid or solid) in a closed vial [42]. Although this technique has been used firstly at the end of the 1980s for the determination of K_f_ values for volatiles with CD [43], its development really began at the end of the 1990s with the work of Sanemasa and Saito [44,45]. Afterwards, this technique has been widely applied to determine K_f_ values for CD/aroma inclusion complexes [8,9,17].

Different treatments were successively developed for the determination of K_f_ values. The first SH-GC methods required a calibration curve to evaluate the concentration of the free guest in the presence of CD. The method developed by Saito [45] used a fixed CD concentration and various concentrations of the volatile guest.

(2) $$\left[G\right]=\frac{{\left[G\right]}_{T}}{{1+K}_{f}\;\left[\mathrm{CD}\right]}$$

with [G]_T_ the initial guest concentration and [G] the free guest concentration. [G]_T_ is known and [G] could be obtained from the calibration curve.

In the meantime, Sanemasa’s group [46] developed a method using a fixed guest concentration and different CD concentrations. The plot of A_0_/A_CD_ versus [CD] gives a straight line, the slope of which corresponds to the K_f_ value:

(3) $$\left[\mathrm{CD}\right]={\left[\mathrm{CD}\right]}_{T}-\frac{\left(A_{0}{-A}_{\mathrm{CD}}\right)}{K^{\prime }}$$

where [CD]_T_ is the initial CD concentration, [CD] is the free CD concentration, A_0_ is the chromatographic peak area of the volatile guest in water (absence of CD), A_CD_ stands for the peak area in the presence of CD and K’ is the slope of the calibration curve.

In 2007, Fourmentin et al. [47] proposed a method that did not require a calibration curve. In this method, a fixed guest concentration and different CD concentrations are used. An algorithmic treatment based on the Equation (4) is used to calculate the K_f_ value from the experimental data [48]:

(4) $$\left[\mathrm{CD}/G\right]=-\frac{1}{2}\sqrt{\left[{\left(\frac{1}{K_{f}}+{\left[\mathrm{CD}\right]}_{T}+{\left[G\right]}_{T}\right)}^{2}-4{\left[\mathrm{CD}\right]}_{T}{\left[G\right]}_{T}\right]}+\frac{1}{2}\left(\frac{1}{K_{f}}+{\left[\mathrm{CD}\right]}_{T}+{\left[G\right]}_{T}\right)$$

The use of gas chromatography allows the quantification but also the separation of volatile compounds. To take benefit from this property, authors have developed methods for the determination of simultaneous K_f_ values. Saito reported the simultaneous determination of the K_f_ of four aromas [49] and six alkanols [50]. This method is based on Equation (2). The guest’s concentration and a calibration curve are required. More recently, Fourmentin et al. [51] have developed a method that precludes the knowledge of the guest’s concentration based on the following equation:

(5) $$K_{f}=\frac{\left(A_{0}{-A}_{\mathrm{CD}}\right)-1}{{\left[\mathrm{CD}\right]}_{T}}$$

where A_0_ and A_CD_ are the chromatographic peak areas of each guest in the absence and the presence of CD, respectively and [CD]_T_ is the initial CD concentration.

This method finds particular usefulness in the determination of K_f_ values of individual components in a complex mixture where the concentration of each component is not known. This is for example the case of essential oils. Kfoury et al. successfully applied this method for the determination of the K_f_ values of aroma compounds with different CD in a wide variety of essential oils [9,52]. Table 2 represents the data collected from the literature on the determination of K_f_ values using SH-GC.

#### 2.2.2. High-Performance Liquid Chromatography

The stability of inclusion complexes is also studied by high-performance liquid chromatography (HPLC). The application of HPLC to evaluate K_f_ values requires the modification of the system. Generally the mobile phase is modified with CD [61]. This implies that the adsorption of CD on the stationary phase is very weak and, thus, it does not influence its properties. The guest molecule is injected in the system. It is adsorbed at the surface of the stationary phase and encapsulated by CD in the mobile phase. The obtained inclusion complexes are not adsorbed on the stationary phase. The guest that forms the most stable inclusion complex with CD is firstly eluted from the HPLC column [61,62]. The retention factor (R) of the guest is determined as a function of the CD concentration. Then, the K_f_ value is obtained as follows:

(6) $$R=\frac{R_{0}}{1+K_{f}\left[\mathrm{CD}\right]}$$

where R and R_0_ are the retention factor observed in the system with and without the CD, respectively. K_f_ is the formation constant of the inclusion complex and [CD] is the concentration of CD in the mobile phase.

The K_f_ value of the β-CD/camphor inclusion complex was successfully determined using HPLC and was equal to 350 M^−1^ [61]. K_f_ values for β-CD inclusion complexes with geraniol, (+)-terpineol and (−)-terpineol were also assessed. They were equal to 334, 413 and 399 M^−1^, respectively [62].

### 2.3. Spectroscopic Methods

#### 2.3.1. UV-Visible Spectroscopy

The common approach to determine K_f_, using UV-Visible spectroscopy, is the direct titration method. One component of the complex (generally the CD) is gradually added to a fixed concentration of the other component of the system (the guest). Meanwhile, the variation in the absorbance peak of the guest is monitored. The CD are silent (they do not absorb). This reduces the complexity of the analysis. The obtained experimental results (absorbance values) are then compared and fitted to binding models to calculate the K_f_ value. Many researchers still make assumptions based on outdated linear regression methods to determine K_f_. These include Benesi-Hildebrand, Scott and Scatchard plots (Table 3). The assessment of a K_f_ value is based on the examination of the slope and intercept of these plots.

The Benesi-Hildebrand plot was also used in the literature to calculate K_f_ values for several inclusion complexes with volatile guests (Table 4). However, these linear regression approaches frequently involve assumptions. They mainly presume the concentration of CD at equilibrium to be equal to its initial concentration. It is also assumed that the variations in the absorbance are proportional to the complex concentration and that the complex is fully formed (all guest is encapsulated) at the end of titration (high CD concentrations). Thus, typically when performing a direct titration experiment a starting molar ratio of [CD]/[guest] equal to 100 is required to perform accurate evaluation of K_f_ [66].

Some K_f_ values are much higher or lower than the range of most K_f_. This could be related to the wrong application of linear equations [67], the use of low CD concentrations and thus not respecting the experimental conditions of linear regression approaches [68] or the incautious choice of the correct wavelength leading to diffraction phenomena [69]. Also, the inclusion of the guest in the CD cavity might be associated with a bathochromic or a hypsochromic shift of its maximum absorbance wavelength. Thus, the measurements should occur at a precise and unique wavelength for all the spectra.

These linear transformations are now being less exploited in the profit of the non-linear regression. This approach is readily achieved by the power of computer software equipped with an algorithm. The algorithm postulates a K_f_ value and an intrinsic response for the fully complexed species, e.g., absorbance values, and compares them to the experimental results. K_f_ and intrinsic response are varied until the best fit is obtained.

Moreover, a distinguished analytical improvement of the determination of K_f_ values was established by Landy et al. [48]. Authors have used the derivatives of the spectra instead of the absorption curves to calculate the K_f_ value. This avoids the difficulties related to experimental errors, to small spectral variations and to the optical presence of CD. CD could, occasionally, result in very weak values of absorbance.

The development of these algorithmic treatments solved the problems related to making assumptions. However, it cannot beat the experimental pitfalls for the application of titration experiments for CD/volatile guest inclusion complexes; (a) the loss of the volatile compound by evaporation while collecting a large number of experimental points; the constancy of the guest concentration is an essential point when measuring the variations in the absorbance, (b) the potential influences of impurities, (c) the very poor chromophore of volatile compounds and (d) the very poor solubility of the volatile guests, though the concentration chosen must lie within the region where the absorption peak is within the limits of the Beer–Lambert Law.

**Figure 3:**
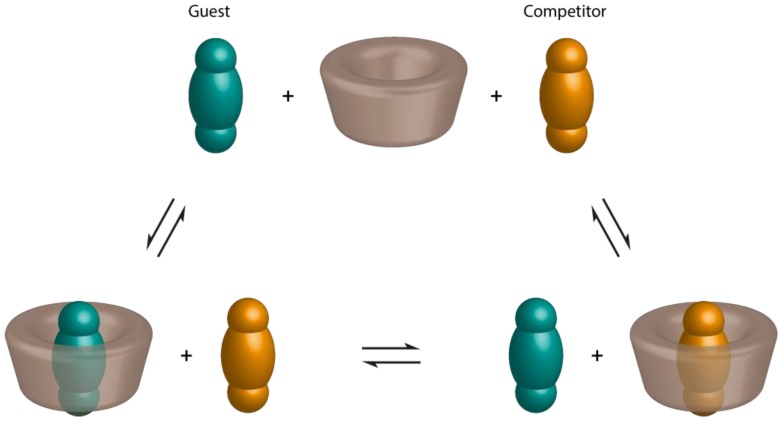
Schematic representation of the spectral displacement method.

Thus, alternative methodologies that can yield more reliable results were developed. Many studies focused on the determination of K_f_ values for CD/guest inclusion complexes by competition with dyes [70]. The competition is monitored by UV-Visible spectroscopy. This method is also called the spectral displacement method. It is not restricted to be applied with a precise dye or CD. Though, the competitive system should be optimized before application. Mainly, the stability (K_f_ value) of the CD/competitor (via a direct titration) should be evaluated. Moreover, to obtain accurate measurements, the selected indicator dye should possess an equal, or higher, K_f_ with CD than that the competing guest [71,72]. The most used dyes, employed as competitors, are the phenolphthalein and the methyl orange. CD reduce the purple phenolphthalein and orange methyl orange solutions upon encapsulation. The addition of the colorless competing guest leads to the restoration of the color by expelling the dye from the CD cavity (Figure 3).

The data collected from the differences between the absorbance values allow the evaluation of K_f_, using an algorithmic treatment. The spectral displacement approach seems to be the method of choice for volatile hydrophobic guests that could not be studied by direct method; (a) the concentration of the guest may be lower than with the titration method, (b) it may be applied to chromophore-less guests, and (c) it reduces the loss by volatilization, since the guest is directly added to the competition system containing the CD, which allows its solubilization and its retention in solution due to its inclusion in the cavity. Therefore, this method has been further applied for CD/volatile guest inclusion complexes. Table 4 summarizes the K_f_ values determined by UV-Visible spectroscopy and collected from the literature.

#### 2.3.2. Fluorescence Spectroscopy

Fluorescence spectroscopy is a useful technique for studying the formation of CD inclusion complexes with fluorescent guests in solution. Due to its high sensitivity, fluorescence spectroscopy allows working with very low guest concentrations [11,82,83]. The CD-induced fluorescence variation allows monitoring encapsulation and quantification of binding strength (K_f_ value) with the fluorescent guest [82].

Generally, titration experiments are carried out. The object is to follow the variation (commonly enhancement) in the fluorescence intensity of the guest as a function of the CD concentration. As in the case of the UV-Visible spectroscopy, the next point is to set an equation that relates the measured fluorescence signal to the total concentration of the CD and the guest. Also, the equations are mostly simplified or transformed to linear equations: Benesi-Hildebrand, Scott or Scatchard.

In addition, any treatment should be only applied at very low absorbance values where the values of the fluorescence intensity vary linearly with the binding magnitude.

The first observation of fluorescence enhancement of volatile guests upon inclusion in CD was reported by Hoshino et al. 1981 [84]. Authors have calculated K_f_ values for β-CD inclusion complexes with benzene, phenol, ethoxybenzene, aniline, *N*-methylaniline, *N*,*N*-dimethyl-aniline, and *N*,*N*-diethylaniline (Table 5). Application of fluorimetric studies has been extended to a wide variety of CD/volatile inclusion complexes. The results are listed in Table 5.

Fluorescence spectroscopy could be considered as a suitable alternative to UV-Visible spectroscopy due to its lower detection limit. Moreover, the fluorescence intensity enhancement upon CD encapsulation results in lowering the limits of detection of poorly fluorescent guests [86,87].

Although fluorescence spectroscopy is fast and very sensitive, the preparation of samples is tricky because a strict cautious is required to avoid any interferences [11,83].

Still, most technologically interesting CD/guest inclusion complexes are themselves non-fluorescent. Thus, competitive methods are being developed to enlarge the application of fluorescence spectroscopy, mainly for fluorophore-free guests. CD are able to enhance the fluorescence of 8-anilinonaphthalene-1-sulfonic acid (ANS) and 2-p-toluidinylnaphthalene-6-sulfonate (TNS) upon encapsulation due to the variation of the polarity of the environment of these dyes [88,89]. The addition of a competitive guest to CD/ANS or CD/TNS solutions results in a decrease in the fluorescence as the fluorophore is moved out of the cavity [90]. This variation in the fluorescence intensity allows the determination of the K_f_ value of CD/fluorophore-free guest inclusion complex.

Finally, one must be careful in interpreting and comparing K_f_ values determined using various techniques, especially those evaluated by fluorescence spectroscopy. When applying fluorescence technique, new species, excited state guest, are involved. The latter can bind to CD in a different strength due to the modification of the electrostatic interactions in the complex. This could result in incoherent K_f_ values as compared to other techniques [91].

#### 2.3.3. Nuclear Magnetic Resonance

Nuclear magnetic resonance (NMR) spectroscopy is a powerful tool and is becoming a routine method for the characterisation of CD inclusion complexes. It is mainly employed for the elucidation of the geometric accommodation of the guest inside the CD cavity but also for the determination of the K_f_ value [92]. NMR provides a direct evidence on the inclusion of the guest in the CD. This is based on the fact that, if the guest is encapsulated, then the physical or chemical environment of the guest and cavity hydrogens (H3 and H5 of the CD) will be affected leading to a modification of the NMR spectra [93].

The NMR shift titrations are one of the most used methods to evaluate the K_f_ value. They are based on the measurements of the chemical shift (δ) changes upon varying concentrations of the CD and/or the guest. Interestingly, the concentration of the species responsible for the signal has to remain strictly constant [48]. Landy et al. have calculated the K_f_ values for the inclusion complexes of β-CD and four phenol derivatives, using an algorithmic treatment applied to the chemical shifts variations of the inner hydrogens of β-CD [48]. The K_f_ values of the inclusion complexes of benzoic acid [94] and fenchone [95] with native CD or derivatives were studied using ^1^H NMR. The dependences of chemical shift variation of the guests’ protons versus CD concentration were used for the K_f_ calculation. Also, DOSY titrations were performed and resulted in a K_f_ value equal to 9.8 M^−1^ for β-CD/vanillin inclusion complex [96]. The DOSY titrations relies on the observation of the variantion in the diffusion coefficients (D) of the guest’s protons. The obtained K_f_ values for inclusion complexes studied with NMR spectroscopy are listed in Table 6.

Lately, a new NMR method has been developed and validated. It consists on an algorithmic treatment and a global analysis to determine the K_f_ value [77]. This analysis explores, at the same time, the variation of several signals of the guest’s protons e.g., the chemical shifts (δ) and the diffusion coefficients (D) [14,77]. Furthermore, it combines simultaneously the responses of numerous protons of the guest. Authors, have applied this method successfully for the inclusion complexes with different aromas: carvacrol, thymol and nootkatone (Table 6).

One of the most important advantages of the NMR titrations is that the detected variations reveal at the same time the conformation of the obtained inclusion complex, which is impossible to extract from other spectroscopic, chromatographic or calorimetric methods [92]. However, the NMR spectroscopy experiences also some drawbacks. The K_f_ values obtained in the deuterated solvents e.g., D_2_O are slightly different than in water. Also, deuterated solvents are generally provided in small amount, leading to errors in the concentration calculations when compared to water [48].

### 2.4. Isothermal Titration Calorimetry

Isothermal Titration Calorimetry (ITC) is the only technique which gives access to both K_f_ values and additional thermodynamic data, in a simultaneous way [98]. Indeed, the injection of one of the CD/guest partners on the other induces a heat release (or more rarely, a heat consumption) which is directly proportional to both inclusion stability and enthalpy (ΔH). As a result, recording the time dependence of the differential power applied to the measuring cell leads to a thermogram, from which a binding isotherm can be extracted. This isotherm corresponds to the heat as a function of species concentrations, generally expressed as a molar ratio. A full algorithmic treatment can then be employed to minimize the difference between experimental and theoretical isotherms, affording the most probable values of K_f_ and ΔH, according to the postulated binding model. It is then straightforward to derive binding free enthalpy (ΔG) and entropy (ΔS). Within this scope, it is noteworthy to mention that thermodynamic data derived from linear van’t Hoff plots (ln K_f_ versus l/T) are generally biased [99], since heat capacity variations of CD complexes are usually different from zero, thus leading to a temperature dependence of both inclusion enthalpy and entropy. In this respect, calorimetric approaches should be considered as the only tool able to afford a reliable thermodynamic picture of inclusion complexes. Finally, non-conventional ITC experiments might be used when classical titrations failed to afford accurate results [100].

K_f_ values, inclusion enthalpy and entropy obtained for the complexes formed between volatile guests and native α-CD or β-CD are summarized in Table 7. If most complexes correspond to 1:1 stoichiometry, linear chains with more than 7 carbons or bulky molecules like camphor can also involve 2:1 inclusion complexes, especially with α-CD [101,102].

If a wide range of enthalpy and entropy variation is observed, the mean values of ΔH and −TΔS are respectively equal to −19.1 and 4.4 kJ/mol for α-CD, and to −9.1 and −8.3 kJ/mol for β-CD. Accordingly, on an averaged point of view, while enthalpy and entropy positively and equally contribute to β-CD complexation, inclusion within α-CD is essentially enthalpy driven, entropy being weakly disfavorable. Such results could be anticipated from the respective size of α-CD and β-CD: the narrower α-CD should lead to more constrained inclusion structures, which imply stronger interactions between CD and guest but also less freedom between the two partners. Within this scope, if no enthalpy/entropy compensation can be established for all guests included in Table 7, such trend clearly appears for guests with homogeneous structures, as pointed out by [103,104]. For instance, the complexation occurring between α-CD and linear alkanols is characterized by a strong correlation between the ΔH and −TΔS components (R^2^ equal to 0.84, if the values of Fujisawa, which are unusually high, are excluded), thus reasserting the opposite character of interaction and freedom upon inclusion.

In addition, the negative ΔH values recorded for most complexes may constitute a valuable proof of the prominent influence of direct interactions (van der Waals, hydrogen bonds) on the complex stability. The fact that entropy may also favor the inclusion process demonstrates that hydrophobic forces also represent a part of the inclusion stabilization.

Many articles [101,102,105] demonstrated that inclusion complexes are characterized by significant negative heat capacity variations (ΔCp), which substantiates the strong influence of solvatophobic effects. The observed increase in affinity and in favorable entropy when moving from H_2_O to D_2_O also pleads in favor of the solvent organization as a driving force for inclusion [102,104]. Finally, analogous conclusions can be drawn from the salt effect [108,114].

### 2.5. Phase Solubility Studies

**Figure 4:**
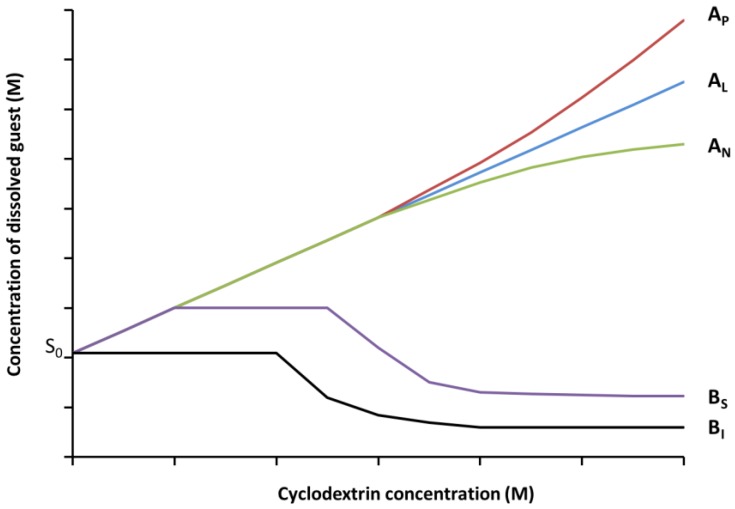
Phase solubility profiles and classification of inclusion complexes according to Higuchi and Connors [115].

Solubility measurements are performed according to the method developed by Higuchi and Connors, 1965 [115]. Excess amounts of guest are added to aqueous solutions containing various concentrations of CD and agitated until equilibrium. Thereafter, the solutions are filtered and the amount of the solubilized guest could be determined using various analytical methods (HPLC, UV-Visible, SH-GC, etc.). Phase solubility diagrams (Figure 4) are obtained by plotting the solubility of the guest as a function of the CD concentration.

The K_f_ values could be obtained from the linear part of the phase solubility diagram.

(7) $$K_{f}=\frac{\mathrm{slope}}{S_{0}\left(1-\mathrm{slope}\right)}$$

where S_0_ is the solubility of the guest in the absence of CD, slope is the slope of the phase solubility diagram.

This method is widely used for the determination of the K_f_ of CD/guest inclusion complexes. In the case of volatile guests, it was mainly applied to fragrance materials and in a lesser extend to anesthetics and organic volatile compounds. Table 8 gathers some K_f_ values obtained with HP-β-CD for different guests and using different analytical methods.

The fact that the presence of CD has an impact on the physicochemical properties of the guest (e.g., absorption, volatility) results in the necessity to dilute the solutions obtained after filtration to cancel this effect and determine the accurate guest concentration. This dilution is often omitted in the phase solubility studies or conducted using an inappropriate solvent leading to wrong K_f_ determination.

### 2.6. Total Organic Carbon

Total organic carbon (TOC) is mainly used in monitoring water quality or cleanliness of pharmaceutical manufacturing equipment [121]. Recently, a new TOC method was developed and applied for studying CD inclusion complexes [36]. Authors have performed TOC measurements to determine the amount of solubilized guest in the filtrates of phase solubility studies. No significant differences were observed for K_f_ values obtained for HP-β-CD/eugenol inclusion complex using UV-Visible spectroscopy (416 M^−1^) [75] and TOC measurements (481 M^−1^) [36]. Authors have also conducted successfully phase solubility studies for eleven essential oils.

The TOC method is interesting for volatiles that generally do not possess a chromophore or a fluorophore moiety because it is non-specific. This technique can be also applied to any molecule that cannot be studied by conventional techniques, making it a universal method for any compound or mixture of compounds. However, this cannot lead to the determination of K_f_ values in the case of mixtures.

### 2.7. Comparison of Formation Constants Obtained with Differents Methods

Some of the volatile guests were studied with various methods. Table 9 gathered some of the K_f_ values obtained for their inclusion complexes using various techniques. As can be seen, for some of the guests (e.g., benzene and toluene) there is a good correlation between the data even though values were obtained with a gap of 20 years. However, for eugenol, we can notice a 10^4^ factor between values obtained with UV-Visible spectroscopy. If we compare values obtained with different methods we can conclude that the K_f_ value for β-CD/eugenol inclusion complex is in the order of 10^2^ M^−1^. This order of magnitude is in good agreement with values for other aromatic derivatives. Therefore, there is a need to determine K_f_ values with accurate analytical protocols in order to obtain reliable results. Moreover, the order of magnitude of some well-known structure (aromatic ring, adamantane derivatives, cyclic compounds) should be a reference for authors.

Concerning the various analytical methods used for the determination of K_f_ values of volatile guests, SH-GC seems to be the method of choice, because it analyses directly the signal of the guest without any interference of the CD signal.

## 3. Conclusions

CD are one of the most appropriate encapsulation materials for volatile guests. The great interest and the advantages of the use of CD inclusion complexes have been widely discussed and documented in the literature. The analytical characterization of the inclusion complex is crucial to best exploit the potential offered by CDs to the encapsulated volatile guests. A careful determination of the K_f_ value of a CD inclusion complex represents the basic fundamental step. This allows the extraction of valuable information concerning CD/guest interactions and strength of binding. However, for some compounds the literature data show very different results. These errors are mainly related to the misuse of the analytical methods and the non-respect of analytical conditions. Therefore, the determination of K_f_ values with different methods or the comparison of the literature data could give a good estimation of the order of magnitude of these K_f_ values.

Spectroscopic, chromatographic and calorimetric techniques have played and still play an important role for this purpose. Titration experiments are usually carried out using linear methods and this apparently simple method seems to be the main source of error. Therefore, one has to be careful when determining K_f_ value because this step is more complex that it seems to be.

This review presented an overview of the analytical techniques and methods applied for the determination of K_f_ values for CD/volatile guest inclusion complexes. It has focused on the advantages, pitfalls and obtained results of each. It also emphasized the search for improving these available methods and developing new techniques in order to have a panel fitting quite all the experimental cases.

## Figures and Tables

**Table 1:** Formation constants (K_f_, M^−1^) for benzene and alkylbenzenes [38].

Guest	α-CD	β-CD	γ-CD
Benzene	17	120	12
Toluene	33	140	20
Ethylbenzene	110	330	36
*o*-Xylene	22	300	34
*m*-Xylene	40	160	27
*p*-Xylene	72	240	8

**Table 2:** Formation constants (K_f_, M^−1^) for CD inclusion complexes with volatile guests obtained by static headspace-gas chromatography (SH-GC).

Guest	α-CD	β-CD	γ-CD	CRYSMEB	RAMEB	HP-β-CD	SBE-β-CD
*trans*-Anethole	710 ^a^ 1163 ^a^	497 ^a^ 630 ^a^ 1040 ^a^	96 ^a^	877 ^a^ 740 ^a^	1110 ^a^ 1553 ^a^	981 ^a^ 1042 ^a^	-
Benzene	19 ^b^ 20 ^c^	128 ^d^ 111 ^c^	9 ^c^	-	110 ^c^	139 ^e^ 94 ^d^ 99 ^c^	-
Benzyl acetate	-	-	-	-	-	230 ^f^	124 ^g^
Benzyl alcohol	52 ^h^	64 ^h^		56 ^h^	53 ^h^	63 ^h^	12 ^g^
1-Butanol	73 ^i^ 74 ^j^ 81 ^k^	14 ^k^	2 ^k^	-	-	-	-
iso-Butylbenzene	-	-	-	-	-	7665 ^l^	-
n-Butylbenzene	-	-	-	-	-	3410 ^l^	-
*tert*-Butylbenzene	-	9503 ^d^	-	-	-	8224 ^l^ 1863 ^d^	-
*tert*-Butylcyclohexane	-	4092 ^d^	-	-	-	2036 ^d^	-
Camphene	598 ^a^	4825 ^a^	360 ^a^	6625 ^a^	6057 ^a^	3033 ^a^	-
Camphor	184 ^a^	2058 ^a^	1048 ^a^	1901 ^a^	1194 ^a^	1280 ^a^	
Carbon tetrachloride	40 ^m^	164 ^m^	-	215 ^m^	238 ^m^	218 ^m^	-
β-Caryophyllene	-	28,674 ^a^	4004 ^a^	11,488 ^a^	14,274 ^a^	4960 ^a^	-
Chloroform	34 ^m^	60 ^m^	-	55 ^m^	93 ^m^	61 ^m^	-
Cinnamaldehyde	236 ^h^	450 ^h^	-	595 ^h^	1696 ^h^	969 ^h^	-
Citronellol	223 ^h^	3141 ^h^	-	3290 ^h^	4048 ^h^	3290 ^f^ 2578 ^h^	-
Cyclohexane	164 ^c^	468 ^c^ 341 ^d^	<10 ^c^	-	474 ^c^	363 ^c^ 227 ^d^	-
*p*-Cymene	140 ^a^	2505 ^a^	88 ^a^	2549 ^a^	3543 ^a^	2213 ^a^	-
Dichloromethane	21 ^m^	9 ^m^	-	9 ^m^	12 ^m^	10 ^m^	-
Ethylbenzene	131 ^c^	392 ^d^ 289 ^c^	125 ^c^	-	320 ^c^	303 ^d^ 248 ^c^	-
Ethylcyclohexane	2017 ^c^	646 ^c^	8 ^c^	-	738 ^c^	630 ^c^	-
Eucalyptol	13 ^a^	615 ^a^	742 ^a^	688 ^a^	673 ^a^	334 ^a^	-
Eugenol	94 ^h^	264 ^h^	-	454 ^h^	568 ^h^	270 ^f^ 462 ^h^	-
Estragole	478 ^a^	939 ^a^	-	1661 ^a^	1761 ^a^	1581 ^a^	-
Geraniol	90 ^h^	528 ^h^	-	977 ^h^	1100 ^h^	1340 ^f^ 712 ^h^	-
1-Heptanol	1493 ^j^ 1586 ^n^ 2460 ^k^	985 ^k^	37 ^k^	-	-	-	-
1-Hexanol	935 ^j^ 509 ^n^ 860 ^k^	260 ^k^	13 ^k^	-	-	-	-
Isoeugenol	85 ^a^	225 ^a^	-	263 ^a^	514 ^a^	441 ^a^	-
*cis*-Jasmone	-	-	-	-	-	1020 ^f^	-
Lilial	4387 ^a^	56,567 ^a^	-	147,617 ^a^	166,338 ^a^	112,205 ^a^	-
Limonene	1289 ^o^	3162 ^o^	116 ^o^ 70 ^p^	3668 ^o^	4386 ^o^	4630 ^f^ 5630 ^q^ 2787 ^o^	4125 ^p^
Linalool	32 ^o^	366 ^o^	138 ^o^	816 ^o^	833 ^o^	940 ^f^ 596 ^o^	-
Linalyl acetate	-	-	-	-	-	1330 ^f^	-
Menthol	82 ^a^	1731 ^a^	105 ^a^	2396 ^a^	1928 ^a^	1079 ^a^	-
Menthone	35 ^a^	656 ^a^	83 ^a^	989 ^a^	748 ^a^	664 ^a^	-
Methylcyclohexane	141 ^c^	332 ^c^ 295 ^d^	9 ^c^	-	374 ^c^	253 ^c^ 202 ^d^	-
Methyl heptine carbonate	2905 ^a^	226 ^a^	-	539 ^a^	485 ^a^	325 ^a^	-
α-iso-Methylionone	71 ^h^	9869 ^h^	-	15,632 ^h^	13,176 ^h^	5750 ^f^ 9789 ^h^	-
Myrcene	212 ^o^	1431 ^o^	138 ^o^ 126 ^p^	959 ^o^	1286 ^o^	1290 ^f^ 1360 ^q^ 575 ^o^	1116 ^p^
1-Nonanol	13,400 ^k^	4900 ^k^	141 ^k^	-	-	-	-
*cis*-Ocimene	42 ^a^	432 ^a^	20 ^a^	622 ^a^	593 ^a^	538 ^a^	-
*trans*-Ocimene	46 ^a^	538 ^a^	26 ^a^	789 ^a^	640 ^a^	627 ^a^	-
1-Octanol	2532 ^j^ 4820 ^k^	1910 ^k^	67 ^k^	-	-	-	-
1-Pentanol	302 ^i^ 286 ^k^ 188 ^n^ 291 ^k^	61 ^k^	3 ^k^	-	-	-	-
2-Pentanol	115 ^k^	25 ^k^	3 ^k^	-	-	-	-
α-Pinene	1778 ^o^	2588 ^o^	214 ^o^ 217 ^p^	2999 ^o^	2395 ^o^	5400 ^f^ 4750 ^q^ 1637 ^o^	1892 ^p^
β-Pinene	1018 ^o^	4587 ^o^	633 ^o^ 404 ^p^	5141 ^o^	4450 ^o^	6650 ^f^ 7070 ^q^ 3151 ^o^	4904 ^p^
Pulegone	30 ^a^	332 ^a^	82 ^a^	1025 ^a^	796 ^a^	676 ^a^	-
Sabinene hydrate	108 ^a^	2108 ^a^	708 ^a^	1308 ^a^	1882 ^a^	772 ^a^	-
Sevoflurane	18 ^r^	150 ^r^	9 ^r^	-	-	163 ^r^	-
γ-Terpinene	37 ^a^	1309 ^a^	40 ^a^	1950 ^a^	2066 ^a^	1488 ^a^	-
α-Terpineol	126 ^a^	1143 ^a^	89 ^a^	1223 ^a^	1287 ^a^	761 ^a^	-
Thymol	^-^	-	-	-	-	806 ^a^	-
Toluene	36 ^b^ 38 ^s^ 29 ^c^	142 ^s^ 158 ^d^ 172 ^c^	33 ^c^	-	171 ^s^ 144 ^c^	182 ^e^ 163 ^s^ 131 ^d^ 170 ^c^	-
*o*-Xylene	18 ^b^ 10 ^c^	184 ^c^	57 ^c^	-	225 ^c^	263 ^e^ 187 ^c^	-
*m*-Xylene	60 ^b^ 36 ^c^	100 ^c^	18 ^c^	-	216 ^c^	222 ^e^ 167 ^c^	-
*p*-Xylene	124 ^b^ 132 ^c^	218 ^c^	32 ^c^	-	300 ^c^	323 ^e^ 236 ^c^	-

CD: cyclodextrin; CRYSMEB: low methylated-β-cyclodextrin; RAMEB: randomly methylated-β-cyclodextrin; HP-β-CD: 2-hydroxypropyl-β-cyclodextrin; SBE-β-CD: sulfobutylether-β-cyclodextrin. ^a^ [2], ^b^ [45], ^c^ [53], ^d^ [51], ^e^ [54], ^f^ [55], ^g^ [43], ^h^ [49], ^i^ [56], ^j^ [57], ^k^ [44], ^l^ [58], ^m^ [47], ^n^ [50], ^o^ [17], ^p^ [52], ^q^ [49], ^r^ [59], ^s^ [60].

**Table 3:** Summary of the equations of linear regression methods.

Method	Equation
Benesi-Hildebrand ^a^	$\frac{1}{\Delta_{i}}=\frac{1}{K_{f}\times \Delta_{\max}\times {\left[\mathrm{CD}\right]}_{i}}+\frac{1}{\Delta_{\max}}$
Scott ^b^	$\frac{{\left[\mathrm{CD}\right]}_{i}}{\Delta_{i}}=\frac{1}{K_{f}}+{\left[\mathrm{CD}\right]}_{i}$
Scatchard ^c^	$\frac{\Delta_{i}}{{\left[\mathrm{CD}\right]}_{i}}=-K_{f}\times \Delta_{i}+K_{f}\times \Delta_{\max}$

^a^ [63], ^b^ [64], ^c^ [65]. Δ_i_ stands for the experimental variation of the guest absorbance upon CD addition. Δ_max_ is the experimental variation of the guest absorbance when it is totally encapsulated. [G] is the concentration of the guest. [CD]_i_ is the concentration of CD at each titration point.

**Table 4:** Formation constant (K_f_, M^−1^) values of CD/volatile guest inclusion complexes determined by UV-Visible spectroscopy.

Guest	Method	α-CD	β-CD	γ-CD	CRYSMEB	RAMEB	HP-β-CD
Acetophenone	BH	140 ^a^	188 ^a^	-	-	-	-
Allyl isothiocyanate	SD	-	36 ^b^	-	-	-	-
*trans*-Anethole	SD	927 ^c^	542 ^d^	-	1039 ^c^	1815 ^c^	845 ^d^
Anisole	BH	-	209 ^a^	-	-	-	-
Anisyl alcohol	SD	-	107 ^e^	-	130 ^e^	125 ^e^	156 ^e^
Benzaldehyde	BH	102 ^a^	150 ^a^	-	-	-	-
Benzene	BH	29 ^a^	194 ^a^	-	-	-	-
Benzoic acid	BH	-	120 ^f^	-	-	-	-
Benzonitrile	BH	78 ^a^	170 ^a^	-	-	-	-
Benzyl alcohol	BH	96 ^a^	143 ^a^	-	-	-	-
	SD		63 ^e^	-	57 ^e^	55 ^e^	54 ^e^
Benzyl chloride	BH	204 ^a^	280 ^a^	-	-	-	-
Bromobenzene	BH	540 ^a^	322 ^a^	-	-	-	-
Camphor	-	3 ^g^	19 ^g^	-	-	-	-
Carvacrol	SD	454 ^h^	2620 ^h^	999 ^h^	2421 ^h^	3564 ^h^	2154 ^h^
Chlorobenzene	BH	112 ^a^	196 ^a^	-	-	-	-
Cineole	-	6 ^g^	29 ^g^	-	-	-	-
Citral	-	8 ^g^	31 ^g^	-	-	-	-
3,4-Dimethoxy benzaldehyde	BH	98 ^i^	157 ^i^	-	-	-	-
*N*,*N*-Dimethylaniline	BH	172 ^a^	252 ^a^	-	-	-	-
Estragole	SD	335 ^j^	987 ^j^	52 ^j^	1584 ^j^	1916 ^j^	1508 ^j^
Ethyl benzoate	BH	361 ^a^	539 ^a^	-	-	-	-
Ethyl phenyl ether	BH	171 ^a^	308 ^a^	-	-	-	-
*N*-Ethyl aniline	BH	128 ^a^	217 ^a^	-	-	-	-
Ethylbenzene	BH	104 ^a^	389 ^a^	-	-	-	-
Eugenol	BH	4.95 × 10^4 k^ 10,633 ^l^	3.96 × 10^5 k^	1.47 × 10^5 k^	-	-	4555 ^l^
	-	5 ^g^	23 ^g^	-	-	-	-
	SD	350 ^c^	322 ^e^	-	401 ^e^	521 ^e^	445 ^e^
Fluorobenzene	BH	39 ^a^	91 ^a^	-	-	-	-
Furaneol	-	1.1 ^g^	7 ^g^	-	-	-	-
Geraniol	BH		51 ^m^	-	-	-	-
	-	9 ^g^	44 ^g^	-	-	-	-
4-Hydroxy-3,5-dimethoxy benzaldehyde	BH	269 ^i^	372 ^i^	-	-	-	-
*p-*Hydroxybenzaldehyde	BH		72 ^n^	-	-	-	-
Iodobenzene	BH	1200 ^a^	846 ^a^	-	-	-	-
Isoeugenol	SD	178 ^c^	304 ^e^	-	240 ^e^	547 ^e^	452 ^e^
Limonene	-	14 ^g^	55 ^g^	-	-	-	-
Menthol	-	10 ^g^	35 ^g^	-	-	-	-
*N*-Methylaniline	BH	83 ^a^	131 ^a^	-	-	-	-
Methyl benzoate	BH	213 ^a^	317 ^a^	-	-	-	-
Methyl cinnamate	-	4 ^g^	20 ^g^	-	-	-	-
Nerol	BH	-	26 ^m^	-	-	-	
Nerolidol	SD	-		-	-	-	8168 ^o^
Nitrobenzene	BH	89 ^a^	279 ^a^	-	-	-	-
Nootkatone	-	7 ^g^	32 ^g^	-	-	-	-
*N*-Phenylacetamide	BH	103 ^a^	157 ^a^	-	-	-	-
Phenol	BH	40 ^a^	95 ^a^	-	-	-	-
Phenylacetylene	BH	86 ^a^	230 ^a^	-	-	-	-
Phenylamine	BH	15 ^a^	86 ^a^	-	-	-	-
Sabinene hydrate	SD	108 ^p^	2108 ^p^	708 ^p^	1308 ^p^	1882 ^p^	772 ^p^
α-Terpineol	SD	126 ^p^	1143 ^p^	89 ^p^	1223 ^p^	1287 ^p^	761 ^p^
Thymol	SD	107 ^h^	1467 ^h^	233 ^h^	2386 ^h^	3337 ^h^	488 ^h^
Toluene	BH	36 ^a^	214 ^a^	-	-	-	-
*o*-Vanillin	BH	105 ^i^	250 ^i^	-	-	-	-
Vanillin	BH	163 ^i^	100 ^n^ 296 ^i^	-	-	-	-
	-	1.6 ^g^	17 ^g^	-	-	-	-
*p*-Vinyl guaiacol	-	2 ^g^	17 ^g^	-	-	-	-

CD: cyclodextrin; CRYSMEB: low methylated-β-cyclodextrin; RAMEB: randomly methylated-β-cyclodextrin; HP-β-CD: 2-hydroxypropyl-β-cyclodextrin; SBE-β-CD: sulfobutylether-β-cyclodextrin; BH: Benesi-Hildebrand; SD; spectral displacement. ^a^ [73], ^b^ [74], ^c^ [75], ^d^ [20], ^e^ [13], ^f^ [76], ^g^ [67], ^h^ [77], ^i^ [78], ^j^ [52], ^k^ [69], ^l^ [68], ^m^ [79], ^n^ [14], ^o^ [80], ^p^ [81].

**Table 5:** Formation constant (K_f_, M^−1^) values for cyclodextrin/volatile guest inclusion complexes determined by fluorescence spectroscopy.

Guest	Method	α-CD	β-CD	HP-β-CD
Aniline	SC	-	50 ^a^	-
Benzene	SC	-	196 ^a^	-
Cinnamaldehyde	BH	-	-	928 ^b^
3,4-Dimethoxybenzaldehyde	BH	124 ^c^	343 ^c^	-
*N*,*N*-Diethylaniline	SC	-	862 ^a^	-
*N*,*N*-Dimethylaniline	SC	-	217 ^a^	-
Ethoxybenzene	SC	-	286 ^a^	-
Eucalyptol	BH	-	-	1200 ^d^
	SC	-	-	1112 ^d^
Eugenol	BH	-	357 ^e^	420 ^e^
Geraniol	BH	-	-	1320 ^d^
	SC	-	-	1064 ^d^
4-Hydroxy-3,5-dimethoxy benzaldehyde	BH	373 ^c^	493 ^c^	-
Limonene	BH	-	-	1667 ^d^
	SC	-	-	1700 ^d^
Linalool	BH	-	-	1500 ^d^
	SC	-	-	1260 ^d^
*N*-Methylaniline	SC	-	53 ^a^	-
Phenol	SC	-	40 ^a^	-
α-Pinene	BH	-	-	2000 ^d^
	SC	-	-	1842 ^d^
β-Pinene	BH	-	-	1667 ^d^
	SC	-	-	1671 ^d^
Pulegone	BH	-	-	867 ^d^
	SC	-	-	798 ^d^
Thymol	BH	-	-	1400 ^d^
	SC	-	-	1313 ^d^
Vanillin	BH	295 ^c^	384 ^c^	-
*o*-Vanillin	BH	183 ^c^	320 ^c^	-

CD: cyclodextrin; HP-β-CD: 2-hydroxypropyl-β-cyclodextrin; BH: Benesi-Hildebrand; SC: Scatrchard. ^a^ [84], ^b^ [15], ^c^ [77], ^d^ [19], ^e^ [85].

**Table 6:** Formation constant (K_f_, M^−1^) values of cyclodextrin/volatile guest inclusion complexes determined by NMR spectroscopy.

Guest	Observed Signal	α-CD	β-CD	γ-CD	HP-α-CD	HP-β-CD	HP-γ-CD
Benzoic acid	δ	842 ^a^	306 ^a^	-	731 ^a^	447 ^a^	551 ^a^
Carvacrol	δ + D	-	1736 ^b^	-	-	-	-
*m*-Cresol	δ	48 ^c^	125 ^c^	97 ^c^	-	130 ^c^	-
(+)-Fenchone	δ	-	550 ^d^	-	-	-	-
(−)-Fenchone	δ	-	523 ^d^	-	-	-	-
Nootkatone	δ + D	-	5750 ^e^	-	-	-	-
Phenol	δ	19 ^c^	60 ^c^	3 ^c^	-	-	-
Thymol	δ + D	-	1344 ^b^	-	-	-	-
Vanillin	D	-	9.8 ^f^	-	-	-	-

CD: cyclodextrin; HP-α-CD: 2-hydroxypropyl-α-cyclodextrin; HP-β-CD: 2-hydroxypropyl-β-cyclodextrin; HP-γ-CD: 2-hydroxypropyl-γ-cyclodextrin; δ: chemical shift; D: diffusion coefficient. ^a^ [94], ^b^ [77], ^c^ [97], ^d^ [95], ^e^ [14], ^f^ [96].

**Table 7:** Formation constants (K_f_, M^−1^), inclusion enthalpy (ΔH, kJ/mol) and entropy (−TΔS, kJ/mol). obtained for the complexes formed between volatiles and native α-CD or β-CD determined by calorimetry.

Guest	α-CD			β-CD		
	K_f_	ΔH	−TΔS	K_f_	ΔH	−TΔS
Benzene	-	-	-	107 ^a^	−3.5	−8.1
(−)-Borneol	-	-	-	19,750 ^b^	−23.2	−1.3
(+)-Borneol	-	-	-	18,640 ^b^	−20.9	−3.5
4-Bromophenol	708 ^c^	−25.6	9.2	851 ^c^	−12.2	−4.5
1-Butanol	83 ^d^	−10.7	−0.2	-	-	-
	100 ^e^	−9.9	−1.5	-	-	-
	80 ^f^	−10.9	0.1	-	-	-
	9153 ^g^	−7.9	−14.7	1430 ^g^	3.0	−21.0
4-Chlorophenol	295 ^c^	−20.1	6	407 ^c^	−11.9	−3.0
Cyclobutanol	63 ^e^	−5.5	−4.8	-	-	-
	30 ^i^	−11.5	3	14 ^i^	3.7	−10.2
Cycloheptanol	-	-	-	2344 ^b^	−11.6	−7.6
	25 ^e^	−32	24	-	-	-
	-	-	-	2200 ^f^	−12.4	−6.7
	68 ^i^	−12.5	2	2197 ^i^	−12.4	−6.7
Cyclohexanol	-	-	-	707 ^b^	−6.1	−10.2
	-	-	-	701 ^f^	−6.3	−9.9
	62 ^j^	−12.8	2.7	776 ^j^	−6.6	−9.8
Cyclooctanol	-	-	-	4425 ^b^	−15.7	−5.1
	28 ^e^	−40	32	-	-	-
	235 ^i^	−3.9	−9.5	4405 ^i^	−16.4	−4.4
Cyclopentanol	-	-	-	168 ^b^	−3.9	−8.8
	-	-	-	175 ^f^	−4.6	−8.2
	83 ^e^	−8.8	−2.1	-	-	-
	36 ^i^	−11.5	2.6	172 ^i^	−4.6	−8.2
Ethanol	7 ^e^	−2.5	−2.2	-	-	-
	7184 ^g^	−0.9	−21.0	1319 ^g^	1.1	−18.9
1-Heptanol	1168 ^d^	−22.8	5.3	-	-	-
	377 ^e^	−20.6	5.9	-	-	-
	24,113 ^g^	−34.6	9.6	17,459 ^g^	−7.9	−16.3
	1503 ^k^	−28.6	10.4	-	-	-
1-Hexanol	705 ^d^	−18.2	1.9	-	-	-
	523 ^e^	−13.9	−1.6	-	-	-
	840 ^f^	−17.5	0.8	-	-	-
	7788 ^g^	−29.1	6.9	5871 ^g^	0.6	−22.1
	906 ^k^	−21.3	4.5	-	-	-
4-Iodophenol	881 ^l^	−26.3	9.5	955 ^l^	−16.1	−0.9
Methanol	0 ^g^	-	-	0 ^g^	-	-
4-Methylphenol	37 ^c^	−17.7	8.6	251 ^c^	−12.5	−1.2
4-Nitrophenol	104 ^l^	−23	11.5	296 ^l^	−10.2	−3.9
	219 ^c^	−25.8	12.5	347 ^c^	−12.0	−2.4
1-Nonanol	480 ^e^	−36.2	20.9	-	-	-
Nootkatone	-	-	-	5801 ^m^	−14.4	−7.1
1-Octanol	220 ^e^	−22.0	9.0	-	-	-
1-Pentanol	246 ^d^	−14.9	1.3	-	-	-
	287 ^f^	−14.7	0.7	-	-	-
	275 ^e^	−11.8	−2.1	-	-	-
	18,927 ^g^	−13.9	−10.4	9153 ^g^	2.2	−24.8
	428 ^k^	−13.8	−1.2	-	-	-
Phenol	37 ^c^	−10.2	0.6	93 ^c^	−12.2	1.2
1-Propanol	23 ^d^	−6.8	−1.0	-	-	-
	27 ^e^	−6.1	−2.1	-	-	-
	1319 ^g^	−6.6	−11.2	1168 ^g^	1.9	−19.4

^a^ [105], ^b^ [106], ^c^ [107], ^d^ [101], ^e^ [108], ^f^ [109], ^g^ [110], ^h^ [102], ^i^ [103], ^j^ [111], ^k^ [112], ^l^ [113], ^m^ [14].

**Table 8:** Formation constant (K_f_, M^−1^) values of HP-β-CD/volatile guest inclusion complexes determined by phase solubility studies using various techniques.

Guest	HPLC	SH-GC	UV-Visible	Sodium Thiosulfate Titration
*trans*-Anethole	-	-	1510 ^a^	-
Benzene	-	-	121 ^b^	-
Benzyl acetate	306 ^c^ 275 ^d^	-	-	-
Carvacrol	-	-	2123 ^e^	-
Citral	1560 ^c^	-	-	-
Citronellol	3740 ^c^	-	-	-
Estragole	-	-	1412 ^a^	-
Ethylbenzene	-	-	435 ^b^	-
Eugenol	-	-	445 ^a^	-
Iodine	-	-	-	38 ^f^
Isoeugenol	-	-	449 ^a^	-
(+)-Limonene	3350 ^c^	4730 ^g^	-	-
Linalool	1610 ^c^ 720 ^d^	940 ^g^	-	-
Linalyl acetate	137 ^c^	-	-	-
α-iso-Methylionone	29,500 ^c^	-	-	-
Myrcene	-	1240 ^g^	-	-
Nootkatone	-	-	3700 ^h^	-
(−)α-Pinene	-	5780 ^g^	-	-
(−)-β-Pinene	-	7360 ^g^	-	-
Propofol	-	-	3972 ^i^	-
Thymol	-	-	1282 ^e^	-
Toluene	-	-	287 ^b^	-
*o*-Xylene	-	-	305 ^b^	-
*m*-Xylene	-	-	210 ^b^	-
*p*-Xylene	-	-	353 ^b^	-

^a^ [75], ^b^ [116], ^c^ [117], ^d^ [118], ^e^ [77], ^f^ [119], ^g^ [21], ^h^ [14], ^i^ [120].

**Table 9:** Example of formation constant (K_f_, M^−1^) values obtained with different methods for some selected guest.

Guest	SH-GC	UV-Visible	Fluorescence	HPLC	ITC	NMR	Phase Solubility
*trans*-Anethole	630 ^a^ 1040 ^a^ 497 ^a^	542 ^b^	^-^	^-^	^-^	^-^	537 ^c^
Benzene	128 ^d^ 111 ^e^	194 ^f^	196 ^g^	-	107 ^h^	-	-
Camphor	2058 ^a^	19 ^i^	-	350 ^j^	-	-	-
Eugenol	264 ^k^	3.96 × 10^5 l^ 23 ^i^ 322 ^m^	357 ^n^	-	-	-	513 ^c^
Limonene	3162 ^a^ 2230 ^a^	55 ^i^	-	-	-	-	-
Phenol	-	95 ^f^	40 ^g^	-	93 ^o^	120 ^p^	-
Thymol	-	1467 ^q^	1400 ^r^	-	-	1344 ^q^	1150 ^q^
Toluene	142 ^s^ 158 ^d^ 172 ^e^	214 ^f^	-	-	-	-	-
Vanillin	-	100 ^t^ 296 ^u^ 17 ^i^	384 ^u^	-	-	9.8 ^v^	-

^a^ [2], ^b^ [20], ^c^ [75], ^d^ [51], ^e^ [53], ^f^ [73], ^g^ [84], ^h^ [105], ^i^ [67], ^j^ [61], ^k^ [49], ^l^ [69], ^m^ [13], ^n^ [85], ^o^ [107], ^p^ [48], ^q^ [77], ^r^ [19], ^s^ [60], ^t^ [14], ^u^ [78], ^v^ [96].

## References

[1] Crini G. (2014). Review: A history of cyclodextrins. Chem. Rev..

[2] Kfoury M., Hadaruga N., Hadaruga D., Fourmentin S. (2016). Cyclodextrins as encapsulation material for flavors and aroma. Encapsulations.

[3] Cabral Marques H.M. (2010). A review on cyclodextrin encapsulation of essential oils and volatiles. Flavour Fragr. J..

[4] Rakmai J., Cheirsilp B., Mejuto J.C., Torrado-Agrasar A., Simal-Gándara J. (2017). Physico-chemical characterization and evaluation of bio-efficacies of black pepper essential oil encapsulated in hydroxypropyl-beta-cyclodextrin. Food Hydrocoll..

[5] Rakmai J., Cheirsil B., Torrado-Agrasar A., Simal-Gándara J., Mejuto J.C. (2017). Encapsulation of yarrow essential oil in hydroxypropyl-beta-cyclodextrin: Physiochemical characterization and evaluation of bio-efficacies. CyTA J. Food.

[6] Rakmai J., Cheirsilp B., Mejuto J.C., Simal-Gándara J., Torrado-Agrasar A. (2018). Antioxidant and antimicrobial properties of encapsulated guava leaf oil in hydroxypropyl-beta-cyclodextrin. Ind. Crops Prod..

[7] Ciobanu A., Mallard I., Landy D., Brabie G., Nistor D., Fourmentin S. (2012). Inclusion interactions of cyclodextrins and crosslinked cyclodextrin polymers with linalool and camphor in *Lavandula angustifolia* essential oil. Carbohydr. Polym..

[8] Decock G., Landy D., Surpateanu G., Fourmentin S. (2008). Study of the retention of aroma components by cyclodextrins by static headspace gas chromatography. J. Incl. Phenom. Macrocycl. Chem..

[9] Kfoury M., Auezova L., Greige-Gerges H., Fourmentin S. (2015). Promising applications of cyclodextrins in food: Improvement of essential oils retention, controlled release and antiradical activity. Carbohydr. Polym..

[10] Reineccius T.A., Reineccius G.A., Peppard T.L. (2003). Flavor release from cyclodextrin complexes: Comparison of alpha, beta, and gamma types. J. Food Sci..

[11] Mura P. (2014). Analytical techniques for characterization of cyclodextrin complexes in aqueous solution: A review. J. Pharm. Biomed. Anal..

[12] Astray G., Gonzalez-Barreiro C., Mejuto J.C., Rial-Otero R., Simal-Gándara J. (2009). A review on the use of cyclodextrins in foods. Food Hydrocoll..

[13] Decock G., Fourmentin S., Surpateanu G.G., Landy D., Decock P., Surpateanu G. (2006). Experimental and theoretical study on the inclusion compounds of aroma components with β-cyclodextrins. Supramol. Chem..

[14] Kfoury M., Landy D., Ruellan S., Auezova L., Greige-gerges H., Fourmentin S. (2017). Nootkatone encapsulation by cyclodextrins: Effect on water solubility and photostability. Food Chem..

[15] Chen H., Ji H., Zhou X., Wang L. (2010). Green synthesis of natural benzaldehyde from cinnamon oil catalyzed by hydroxypropyl-β-cyclodextrin. Tetrahedron.

[16] Jiang S., Li J.N., Jiang Z.T. (2010). Inclusion reactions of β-cyclodextrin and its derivatives with cinnamaldehyde in *Cinnamomum loureirii* essential oil. Eur. Food Res. Technol..

[17] Ciobanu A., Landy D., Fourmentin S. (2013). Complexation efficiency of cyclodextrins for volatile flavor compounds. Food Res. Int..

[18] Ciobanu A., Mallard I., Landy D., Brabie G., Nistor D., Fourmentin S. (2013). Retention of aroma compounds from *Mentha piperita* essential oil by cyclodextrins and crosslinked cyclodextrin polymers. Food Chem..

[19] Kfoury M., Auezova L., Fourmentin S., Greige-Gerges H. (2014). Investigation of monoterpenes complexation with hydroxypropyl-beta-cyclodextrin. J. Incl. Phenom. Macrocycl. Chem..

[20] Kfoury M., Auezova L., Greige-Gerges H., Ruellan S., Fourmentin S. (2014). Cyclodextrin, an efficient tool for *trans*-anethole encapsulation: Chromatographic, spectroscopic, thermal and structural studies. Food Chem..

[21] Tanemura I., Saito Y., Ueda H., Sato T. (1998). Solubility method using static head-space gas chromatography for determination of the stability constants of fragrance materials with 2-hydroxypropyl-β-cyclodextrin. Chem. Pharm. Bull..

[22] Moeder C., O’Brien T., Thompson R., Bicker G. (1996). Determination of stoichiometric coefficients and apparent formation constants for α- and β-CD complexes of terpenes using reversed-phase liquid chromatography. J. Chromatogr. A.

[23] Liu Y., Li B., Wada T., Inoue Y. (2001). Studies on molecular recognition in supramolecular systems. Part 31: Circular dichroism spectral studies of molecular and chiral recognition of aliphatic alcohols by 6-modified β-cyclodextrins. Tetrahedron.

[24] Liu Y., Li L., Zhang H.Y., Yang Y.W., Ding F. (2014). Correlation between thermodynamic behavior and structure in the complexation of modified β-cyclodextrins and bile salts. Supramol. Chem..

[25] Liu Y., Zhang Q., Chen Y. (2007). Spectrophotometric and calorimetric titration studies on molecular recognition of camphor and borneol by nucleobase- modified β-cyclodextrins. J. Phys. Chem. B.

[26] Ansari M.J., Kohli K., Ali J., Anwer M.K., Jamil S., Ahmed M.M. (2014). Physicochemical characterizations and dissolution behavior of curcumin and α-cyclodextrin molecular inclusion complexes. Der Pharm. Lett..

[27] Hill L.E., Gomes C., Taylor T.M. (2013). Characterization of beta-cyclodextrin inclusion complexes containing essential oils (*trans*-cinnamaldehyde, eugenol, cinnamon bark, and clove bud extracts) for antimicrobial delivery applications. LWT Food Sci. Technol..

[28] Karathanos V.T., Mourtzinos I., Yannakopoulou K., Andrikopoulos N.K. (2007). Study of the solubility, antioxidant activity and structure of inclusion complex of vanillin with β-cyclodextrin. Food Chem..

[29] Liang H., Yuan Q., Vriesekoop F., Lv F. (2012). Effects of cyclodextrins on the antimicrobial activity of plant-derived essential oil compounds. Food Chem..

[30] Mazzobre M.F., dos Santos C.I., Buera M. (2011). Solubility and stability of β-cyclodextrin-terpineol inclusion complex as affected by water. Food Biophys..

[31] Mourtzinos I., Kalogeropoulos N., Papadakis S.E., Konstantinou K., Karathanos V.T. (2008). Encapsulation of nutraceutical monoterpenes in β-cyclodextrin and modified starch. J. Food Sci..

[32] Santos E.H., Kamimura J.A., Hill L.E., Gomes C.L. (2015). Characterization of carvacrol beta-cyclodextrin inclusion complexes as delivery systems for antibacterial and antioxidant applications. LWT Food Sci. Technol..

[33] Tao F., Hill L.E., Peng Y., Gomes C.L. (2014). Synthesis and characterization of β-cyclodextrin inclusion complexes of thymol and thyme oil for antimicrobial delivery applications. LWT Food Sci. Technol..

[34] Waleczek K.J., Cabral Marques H.M., Hempel B., Schmidt P.C. (2003). Phase solubility studies of pure (−)-α-bisabolol and camomile essential oil with β-cyclodextrin. Eur. J. Pharm. Biopharm..

[35] Zeng Z., Fang Y., Ji H. (2012). Side chain influencing the interaction between β-cyclodextrin and vanillin. Flavour Fragr. J..

[36] Kfoury M., Auezova L., Greige-Gerges H., Fourmentin S. (2016). Development of a Total Organic Carbon method for the quantitative determination of solubility enhancement by cyclodextrins: Application to essential oils. Anal. Chim. Acta.

[37] Sanemasa I., Kobayashi T., Deguchi T. (1985). Formation constants of cyclodextrin inclusion complexes with iodine in aqueous solutions. Bull. Chem. Soc. Jpn..

[38] Sanemasa I., Fujiki M., Deguchi T. (1988). A new method for determining cyclodextrin complex formation constants with electrolytes in aqueous medium. Bull. Chem. Soc. Jpn..

[39] Osajima T., Deguchi T., Sanemasa I. (1991). Association of cycloalkanes with cyclodextrins. Bull. Chem. Soc. Jpn..

[40] Kolb B. (1999). Headspace sampling with capillary columns. J. Chromatogr. A.

[41] Snow N.H., Slack G.C. (2002). Head-Space analysis in modern gas chromatography. TrAC Trends Anal. Chem..

[42] Kolb B., Ettre L.S. (2006). Static Headspace-Gas Chromatography Theory and Practice.

[43] Qu Q., Tucker E., Christian S.D. (2003). Solubilization of synthetic perfumes by nonionic surfactants and by sulfoalkyl ether β-CDs. J. Incl. Phenom. Macrocycl. Chem..

[44] Wu J.S., Zheng J.Z., Toda K., Sanemasa I. (1999). Association of alcohol-cyclodextrin in aqueous medium determined by headspace gas chromatography. Anal. Sci..

[45] Saito Y. (1997). Determination of the stability constants of benzene and alkylbenzenes with α-cyclodextrin by static head-space gas chromatography. Chem. Pharm. Bull..

[46] Zheng J.Z., Wu J.S., Toda K., Sanemasa I. (2001). Association of n-alcohol with *p*-sulfonato calixarenes in an aqueous medium determined by headspace gas chromatography. Bull. Chem. Soc. Jpn..

[47] Fourmentin S., Outirite M., Blach P., Landy D., Ponchel A., Monflier E., Surpateanu G. (2007). Solubilisation of chlorinated solvents by cyclodextrin derivatives. A study by static headspace gas chromatography and molecular modelling. J. Hazard. Mater..

[48] Landy D., Fourmentin S., Salome M., Surpateanu G. (2000). Analytical improvement in measuring formation constants of inclusion complexes between β-cyclodextrin and phenolic compounds. J. Incl. Phenom..

[49] Saito Y., Tanamura I., Ueda H., Sato T. (1998). Simultaneous determination of the stability constants for fragrance materials with 2-hydroxypropyl-β-cyclodextrin by static headspace gas chromatography. Chem. Pharm. Bull..

[50] Saito Y., Hashizaki K., Taguchi H., Tomono K., Goto H., Ogawa N. (2000). Determination of the stability constants in alkanol/alpha-cyclodextrin mixed system. Drug Dev. Ind. Pharm..

[51] Fourmentin S., Ciobanu A., Landy D., Wenz G. (2013). Space filling of β-cyclodextrin and β-cyclodextrin derivatives by volatile hydrophobic guests. Beilstein J. Org. Chem..

[52] Kfoury M., Pipkin J.D., Antle V., Fourmentin S. (2017). Captisol^®^: An efficient carrier and solubilizing agent for essential oils and their components. Flavour Fragr. J..

[53] Szaniszló N., Fenyvesi É., Balla J. (2005). Structure-stability study of cyclodextrin complexes with selected volatile hydrocarbon contaminants of soils. J. Incl. Phenom. Macrocycl. Chem..

[54] Misawa K., Saito Y., Hashizaki K., Taguchi H., Ogawa N., Ueda H. (2005). Stability constants for 1:1 complexes formed between 2-hydroxypropyl-β-cyclodextrin with an average substitution degree of 4.4 and benzene and alkylbenzenes as guests by modified static head-space gas chromatography method. J. Incl. Phenom. Macrocycl. Chem..

[55] Saito Y., Tanemura I., Sato T., Ueda H. (1999). Interaction of fragrance materials with 2-hydroxypropyl-beta-cyclodextrin by static and dynamic head-space methods. Int. J. Cosmet. Sci..

[56] Hall D., Bloor D., Tawarah K., Wynjones E. (1986). Kinetic and equilibrium studies associated with the formation of inclusion-compounds involving normal-nutanol and normal-pentanol in aqueous cyclodextrin solutions. J. Chem. Soc. Faraday Trans..

[57] Saito Y., Misawa K., Hashizaki K., Taguchi H., Ogawa N., Ueda H. (2004). A modified method using static head-space gas chromatography for determining the stability constants of 1-alkanol/alpha-cyclodextrin complexation. Chem. Pharm. Bull..

[58] Saito Y., Hashizaki K., Taguchi H., Ogawa N. (2003). Complexation of butylbenzenes with 2-hydroxypropyl-cyclodextrins in aqueous solution. J. Environ. Sci. Health Part A Toxic/Hazard. Subst. Environ. Eng..

[59] Becker L.F., Schwarz D.H., Wenz G. (2014). Synthesis of uniform cyclodextrin thioethers to transport hydrophobic drugs. Beilstein J. Org. Chem..

[60] Blach P., Fourmentin S., Landy D., Cazier F., Surpateanu G. (2008). Cyclodextrins: A new efficient absorbent to treat waste gas streams. Chemosphere.

[61] Asztemborska M., Bielejewska A., Duszczyk K., Sybilska D. (2000). Comparative study on camphor enantiomers behavior under the conditions of gas-liquid chromatography and reversed-phase high-performance liquid chromatography systems modified with α- and β-cyclodextrins. J. Chromatogr. A.

[62] Ceborska M., Szwed K., Asztemborska M., Wszelaka-Rylik M., Kicińska E., Suwińska K. (2015). Study of β-cyclodextrin inclusion complexes with volatile molecules geraniol and α-terpineol enantiomers in solid state and in solution. Chem. Phys. Lett..

[63] Hildebrand J.H., Benesi H.A. (1949). Interaction of iodine with aromatic hydrocarbons. J. Am. Chem. Soc..

[64] Scatchard G. (1949). The attractions of proteins for small molecules and ions. Ann. N. Y. Acad. Sci..

[65] Scott R.L. (1956). Some comments on the Benesi-Hildebrand equation. Recl. Trav. Chim. Pays-Bas.

[66] Wang R., Yu Z. (2007). Validity and reliability of Benesi-Hildebrand method. Acta Phys.-Chim. Sin..

[67] Astray G., Mejuto J.C., Morales J., Rial-Otero R., Simal-Gandara J. (2010). Factors controlling flavors binding constants to cyclodextrins and their applications in foods. Food Res. Int..

[68] Hernández-Sánchez P., López-Miranda S., Lucas-Abellán C., Núñez-Delicado E. (2012). Complexation of eugenol (EG), as main component of clove oil and as pure compound, with β- and HP-β–CDs. Food Nutr. Sci..

[69] Yang Y., Song L.X. (2005). Study on the inclusion compounds of eugenol with α-, β-, γ- and heptakis (2,6-di-*O*-methyl)-β-cyclodextrins. J. Incl. Phenom. Macrocycl. Chem..

[70] Pendergast D.D., Connors K.A. (1984). Improved competitive indicator methods for the study of α-cyclodextrin complexes. J. Pharm. Sci..

[71] Sadrerafi K., Moore E.E., Lee M.W. (2015). Association constant of β-cyclodextrin with carboranes, adamantane, and their derivatives using displacement binding technique. J. Incl. Phenom. Macrocycl. Chem..

[72] Selvidge L.A., Eftink M.R. (1986). Spectral displacement techniques for studying the binding of spectroscopically transparent ligands to cyclodextrins. Anal. Biochem..

[73] Guo Q.X., Luo S.H., Liu Y.C. (1998). Substituent effects on the driving force for inclusion complexation of alpha- and beta-cyclodextrin with monosubstituted benzene derivatives. J. Incl. Phenom. Mol. Recognit. Chem..

[74] Zhang Q.F., Jiang Z.T., Li R. (2007). Complexation of allyl isothiocyanate with β-cyclodextrin and its derivatives and molecular microcapsule of allyl isothiocyanate in β-cyclodextrin. Eur. Food Res. Technol..

[75] Kfoury M., Landy D., Auezova L., Greige-Gerges H., Fourmentin S. (2014). Effect of cyclodextrin complexation on phenylpropanoids’ solubility and antioxidant activity. Beilstein J. Org. Chem..

[76] Belyakova L.A., Lyashenko D.Y. (2008). Complex formation between benzene carboxylic acids and beta-cyclodextrin. Appl. Spectrosc..

[77] Kfoury M., Landy D., Ruellan S., Auezova L., Greige-Gerges H., Fourmentin S. (2016). Determination of formation constants and structural characterization of cyclodextrin inclusion complexes with two phenolic isomers: Carvacrol and thymol. Beilstein J. Org. Chem..

[78] Jenita M.J., Mohandass T., Rajendiran N. (2014). Spectral and molecular modeling studies on hydroxybenzaldehydes with native and modified cyclodextrins. J. Fluoresc..

[79] Yang Z.J., Zhou D., Fang Y.X., Ji H.B. (2016). Shape-selective separation of geraniol and nerol via noncovalent interactionswith β-cyclodextrin. Sep. Sci. Technol..

[80] Azzi J., Danjou P.E., Landy D., Ruellan S., Auezova L., Greige-Gerges H., Fourmentin S. (2017). The effect of cyclodextrin complexation on the solubility and photostability of nerolidol as pure compound and as main constituent of cabreuva essential oil. Beilstein J. Org. Chem..

[81] Kfoury M., Balan R., Landy D., Nistor D., Fourmentin S. (2015). Investigation of the complexation of essential oil components with cyclodextrins. Supramol. Chem..

[82] Dsouza R.N., Pischel U., Nau W.M. (2011). Fluorescent dyes and their supramolecular host/guest complexes with macrocycles in aqueous solution. Chem. Rev..

[83] Thordarson P. (2011). Determining association constants from titration experiments in supramolecular chemistry. Chem. Soc. Rev..

[84] Hoshino M., Imamura M., Ikehara K., Hama Y. (1981). Fluorescence enhancement of benzene derivatives by forming inclusion complexes with β-cyclodextrin in aqueous solutions. J. Phys. Chem..

[85] Zhan H., Jiang Z.T., Wang Y., Li R., Dong T.S. (2008). Molecular microcapsules and inclusion interactions of eugenol with β-cyclodextrin and its derivatives. Eur. Food Res. Technol..

[86] Ochocka R.J. (1993). Fluorescence enhancement of two terpenes commonly present in essential oils. J. Pharm. Biomed. Anal..

[87] Uzaşçi S., Erim F.B. (2014). Enhancement of native fluorescence intensity of berberine by (2-hydroxypropyl)-β-cyclodextrin in capillary electrophoresis coupled by laser-induced fluorescence detection: Application to quality control of medicinal plants. J. Chromatogr. A.

[88] Favrelle A., Gouhier G., Guillen F., Martin C., Mofaddel N., Petit S., Mundy K.M., Pitre S.P., Wagner B.D. (2015). Structure-binding effects: Comparative binding of 2-anilino-6-naphthalenesulfonate by a series of alkyl- and hydroxyalkyl-substituted β-cyclodextrins. J. Phys. Chem. B.

[89] Wagner B.D., Fitzpatrick S.J.A. (2000). Comparision of the host-guest inclusion complexes of 1,8-ANS and 2,6-ANS in parent and modified cyclodextrins. J. Incl. Phenom. Macrocycl. Chem..

[90] Divakar S., Maheswaran M.M. (1997). Structural studies on inclusion compounds of β-cyclodextrin with some substituted phenols. J. Incl. Phenom. Mol. Recognit. Chem..

[91] Yañez C., Günther G. (2014). Is the determination of the association constant of cyclodextrin inclusion complexes dependent on the technique. J. Chil. Chem. Soc..

[92] Schneider H.-J., Hacket F., Rüdiger V., Ikeda H. (1998). NMR Studies of cyclodextrins and cyclodextrin complexes. Chem. Rev..

[93] Tsao J., Tsai H., Wu C., Lin P., Su S., Chen L., Tsai F., Tsai Y. (2010). Release of paeonol-β-CD complex from thermo-sensitive poly(N-isopropylacrylamide) hydrogels. Int. J. Pharm..

[94] Terekhova I., Koźbiał M., Kumeev R., Gierycz P. (2011). Complex formation of native and hydroxypropylated cyclodextrins with benzoic acid in aqueous solution: Volumetric and ^1^H NMR study. Chem. Phys. Lett..

[95] Nowakowski M., Ejchart A. (2014). Complex formation of fenchone with α-cyclodextrin: NMR titrations. J. Incl. Phenom. Macrocycl. Chem..

[96] Ferrazza R., Rossi B., Guella G. (2014). DOSY-NMR and raman investigations on the self-aggregation and cyclodextrin complexation of vanillin. J. Phys. Chem. B.

[97] Lantz A.W., Rodriguez M.A., Wetterer S.M., Armstrong D.W. (2006). Estimation of association constants between oral malodor components and various native and derivatized cyclodextrins. Anal. Chim. Acta.

[98] Hansen L.D., Fellingham G.W., Russell D.J. (2011). Simultaneous determination of equilibrium constants and enthalpy changes by titration calorimetry: Methods, instruments, and uncertainties. Anal. Biochem..

[99] Chaires J.B. (1997). Possible origin of differences between Van’t Hoff and calorimetric enthalpy estimates. Biophys. Chem..

[100] Bertaut E., Landy D. (2014). Improving ITC studies of cyclodextrin inclusion compounds by global analysis of conventional and non-conventional experiments. Beilstein J. Org. Chem..

[101] Hallén D., Schön A., Shehatta I., Wadsö I. (1992). Microcalorimetric titration of α-cyclodextrin with some straight-chain alkan-1-ols at 288.15, 298.15 and 308.15 K. J. Chem. Soc. Faraday Trans..

[102] Schmidtchen F.P. (2002). The anatomy of the energetics of molecular recognition by calorimetry: Chiral discrimination of camphor by α-cyclodextrin. Chem. Eur. J..

[103] Rekharsky M.V., Mayhew M.P., Goldberg R.N., Ross P.D., Yamashoji Y., Inoue Y. (1997). Thermodynamic and nuclear magnetic resonance study of the reactions of α- and β-cyclodextrin with acids, aliphatic amines, and cyclic alcohols. J. Phys. Chem. B.

[104] Rekharsky M., Inoue Y. (2000). 1:1 and 1:2 Complexation thermodynamics of γ-cyclodextrin with N-carbobenzyloxy aromatic amino acids and ω-phenylalkanoic acids. J. Am. Chem. Soc..

[105] Gómez-Orellana I., Hallén D. (1993). The thermodynamics of the binding of benzene to β-cyclodextrin in aqueous solution. Thermochim. Acta.

[106] Liu Y., Yang E.C., Yang Y.W., Zhang H.Y., Fan Z., Ding F., Cao R. (2004). Thermodynamics of the molecular and chiral recognition of cycloalkanols and camphor by modified β-cyclodextrins possessing simple aromatic Tethers. J. Org. Chem..

[107] Bertrand G.L., Faulkner J.R., Han S.M., Armstrong D.W. (1989). Substituent effects on the binding of phenols to cyclodextrins in aqueous solution. J. Phys. Chem..

[108] Castronuovo G., Elia V., Iannone A., Niccoli M., Velleca F. (2000). Factors determining the formation of complexes between α-cyclodextrin and alkylated substances in aqueous solutions: A calorimetric study at 25 °C. Carbohydr. Res..

[109] Rekharsky M.V., Inoue Y. (2002). Solvent and guest isotope effects on complexation thermodynamics of α-, β-, and 6-amino-6-deoxy-β-cyclodextrins. J. Am. Chem. Soc..

[110] Fujisawa M., Kimura T. (2004). Enthalpy and entropy changes on molecular inclusion of 1-heptanol into α- and β-cyclodextrin cavities in aqueous solutions. Thermochim. Acta.

[111] Rekharsky M.V., Schwarz F.P., Tewari Y.B., Goldberg R.N., Tanaka M., Yamashoji Y. (1994). Thermodynamic and NMR study of the interactions of cyclodextrins with cyclohexane derivatives. J. Phys. Chem..

[112] Moreira R., Bastos M. (2000). The influence of glycerol on ligand binding equilibria between monoalcohols and α-cyclodextrin. J. Chem. Thermodyn..

[113] Rüdiger V., Eliseev A., Simova S., Schneider H.J., Blandamer M.J., Cullis P.M., Meyer A.J. (1996). Conformational, calorimetric and NMR spectroscopic studies on inclusion complexes of cyclodextrins with substituted phenyl and adamantane derivatives. J. Chem. Soc. Perkin Trans. 2.

[114] Fini P., Castagnolo M., Catucci L., Cosma P., Agostiano A. (2003). The effects of increasing NaCl concentration on the stability of inclusion complexes in aqueous solution. J. Therm. Anal. Calorim..

[115] Higuchi T., Connors K.A. (1965). Phase solubility techniques. Advances in Analytical Chemistry Instrument.

[116] Balogh K., Szaniszló N., H-Otta K., Fenyvesi É. (2007). Can cyclodextrins really improve the selectivity of extraction of BTEX compounds?. J. Incl. Phenom. Macrocycl. Chem..

[117] Matsuda H., Ito K., Fujiwara Y., Tanaka M., Taki A., Uejima O., Sumiyoshi H. (1991). Complexation of various fragrance materials with 2-hydroxypropyl-β-cyclodextrin. Chem. Pharm. Bull..

[118] Numanoglu U., Şen T., Tarimci N., Kartal M., Koo O.M.Y., Önyüksel H. (2007). Use of cyclodextrins as a cosmetic delivery system for fragrance materials: Linalool and benzyl acetate. AAPS PharmSciTech.

[119] Szente L., Fenyvesi É., Szejtli J. (1999). Entrapment of iodine with cyclodextrins: Potential application of cyclodextrins in nuclear waste management. Environ. Sci. Technol..

[120] Trapani G., Lopedota A., Franco M., Latrofa A., Liso G. (1996). Effect of 2-hydroxypropyl-β-cyclodextrin on the aqueous solubility of the anaesthetic agent propofol (2,6-diisopropylphenol). Int. J. Pharm..

[121] Bourgeois W., Burgess J.E., Stuetz R.M. (2001). On-line monitoring of wastewater quality: A review. J. Chem. Technol. Biotechnol..

